# Qualitative Analysis of Barriers and Facilitators to Tobacco Treatment Engagement Among Hispanic Patients with Cancer in New York City

**DOI:** 10.1007/s40615-026-02915-1

**Published:** 2026-04-06

**Authors:** Ruthmarie Hernández-Torres, Jaime Gilliland, Lianel Rosario, Gleneara Bates-Pappas, Lou-Anne Chichester, Rosario Costas-Muñíz, Chris Kotsen, Jamie S. Ostroff

**Affiliations:** 1 Department of Psychiatry and Behavioral Sciences, Memorial Sloan Kettering Cancer Center, New York, USA; 2 School of Behavioral and Brain Science, Ponce Health Science University, Ponce, Puerto Rico

**Keywords:** Hispanic, Cancer, Tobacco Treatment, Barriers, Facilitators

## Abstract

**Background:**

Hispanic patients with cancer who smoke are less likely to quit after diagnosis and underutilize evidence-based tobacco treatment. Understanding the complex factors influencing their engagement is crucial for developing effective and tailored interventions.

**Methods:**

Semi-structured individual interviews were conducted with 12 adult patients with cancer who self-identified as Hispanic or Latino referred between June 1, 2023, and June 30, 2024, to a tobacco treatment program embedded within a cancer center. Interviews were conducted via phone or video in English or Spanish. Transcripts were thematically analyzed, guided by the Socio-ecological Model, assessing individual, interpersonal, organizational, community, and policy barriers and facilitators affecting treatment engagement.

**Results:**

Median age was 65 and most participants (10) were women. Four multilevel themes emerged from the data: (1) Socio-Cultural Drivers and the Psychological Barrier of Fatalism; (2) Provider–Patient Relationship Enhances Engagement; (3) Community and Cultural Contexts Shaping Tobacco Use and Support Needs; and (4) Structural, Economic, And Policy-Level Determinants Constrain Access; Implementation and Enforcement are Key Levers. Additionally, the decision to accept or decline treatment further distinguished by differences in age/fatalism, provider relationship/trust, and the view of willpower versus external support.

**Conclusion:**

Engagement in tobacco treatment among Hispanic patients with cancer is shaped by the intersection of individual, cultural, and structural barriers and facilitators. Findings highlight the need for culturally and linguistically congruent interventions that address fatalism, integrate family support, reduce financial barriers, and leverage the patient-provider relationship to improve access and outcomes.

## Background

Persistent smoking after a cancer diagnosis increases the risk of treatment-related complications, second primary cancers, and other adverse outcomes [1, 2]. In contrast, quitting smoking after a cancer diagnosis is associated with better survival and multiple health benefits, including a lower risk of treatment complications, disease progression, and developing a second primary cancer [3, 4]. Although the risks of smoking and the benefits of quitting following diagnosis are well known, 47.3% of individuals with cancer reported a history of smoking, with 14.7% still smoking at the time of diagnosis in the United States (U.S.) [5]. Hispanic patients with cancer are 33% more likely to report current smoking and are significantly more likely to continue smoking after diagnosis than White patients (RR 1.33; 95% CI: 1.09–1.62; *p* = 0.003) [6]. Furthermore, they are 77% less likely than White patients to quit smoking after diagnosis (OR = 0.23, 95% CI = 0.10–0.57) [7].

In the general population, Hispanic individuals face unique challenges that span multiple levels of the Socio-Ecological Model (SEM) [8–10]. At the individual level, quitting efforts are often hindered by misconceptions about nicotine replacement therapy (NRT) and concerns regarding dependence on pharmacotherapy, alongside higher rates of anxiety and depression particularly among young adults [11–15]. At the organizational and systemic levels, evidence shows that Hispanic patients are historically less likely to be advised to quit by providers or referred to evidence-based treatments [16–20]. Furthermore, community and policy-level factors contribute to a retail environment that normalizes tobacco use, as Hispanic neighborhoods often experience disproportionately high levels of tobacco advertising and price promotions, driven by industry payments to merchants and the strategic sponsorship of cultural events [21, 22]. This culturally targeted marketing exposure through price promotions, menthol product advertising, and community sponsorships further normalizes tobacco use and creates additional barriers to treatment engagement and quitting.

These multilevel challenges are particularly consequential for Hispanics with cancer, who may face compounded barriers in accessing and engaging with tobacco treatment services. In a large cancer care setting, analyses of over 300,000 patients indicate that the prevalence of current tobacco use among Hispanic patients was 6.3%, and acceptance of tobacco treatment among those reached was relatively high (60.5%) [23]. These findings suggest that by embedding tobacco screening and treatment as routine components of high-quality cancer care, opt-out approaches can support equitable access for racially and ethnically diverse patients. Nevertheless, gaps in reach and sustained engagement persist, and Hispanics with cancer who smoke remain underrepresented in cessation trials and often underutilize evidence-based treatments [19]. Understanding multilevel barriers that impede their participation and success in cessation efforts is critical for developing culturally tailored interventions aimed at enhancing treatment engagement and improving health outcomes in this population. Taken together, these observations indicate that Hispanics with cancer who smoke are less likely to engage in tobacco treatment. The current study aims to identify the barriers and facilitators impacting tobacco treatment engagement among Hispanic patients with cancer who smoke and are referred for tobacco treatment services embedded within a large cancer care setting. The findings will advance our understanding of unmet patient needs and guide recommendations for culturally tailored interventions to improve access to tobacco treatment engagement and ultimately reduce tobacco use among Hispanic patients with cancer.

## Methods

### Study Design

This Quality Improvement (QI) study explored the barriers and facilitators of engagement with tobacco treatment among Hispanic patients with cancer who smoke and were referred to a Tobacco Treatment Program (TTP).

### Ethical Review and Patient Engagement

This QI project underwent review by the Memorial Sloan Kettering Cancer Center Institutional Review Board (IRB) and was officially determined not to constitute human subjects research as defined by federal regulations. This classification is appropriate for local QI initiatives focused on improving existing processes. To ensure ethical conduct, all participants were informed about the project and provided verbal consent prior to participation, agreeing to the use of a recording device. Confidentiality and privacy were maintained throughout the project by assigning each participant a sequential identifier (Patient; Pt 1 to Pt 12). Participants retained full autonomy and the ability to withdraw from the project or request clarification at any time.

### Recruitment Process

At Memorial Sloan Kettering Cancer Center (MSK), a leading comprehensive cancer center in New York City, patients are routinely screened for tobacco use as part of standard clinical care. Patients who report current cigarette use are referred to tobacco treatment and may receive up to three follow-up calls to schedule appointments [23]. For this study, eligible patients were identified by querying the electronic patient database to generate a list of potential participants meeting the inclusion criteria. These participants were then contacted by phone to confirm eligibility and assess interest in the study. Interviews were conducted in either English or Spanish according to participant preference to ensure language accessibility. Verbal consent was obtained from all participants prior to data collection.

### Participants

Participating patients were approached consecutively and were eligible for study inclusion if they were aged 18 years or older, had a diagnosis of any type of cancer and were undergoing active treatment, reported smoking cigarettes within the past 30 days and were referred to the MSK TTP within the past 12 months (June 1, 2023 – June 30, 2024). Only patients identified as Hispanic, fluent in either English or Spanish, and with a TTP referral disposition status of either “accepted” or “refused” treatment were included. Of the 612 eligible patients, 71 were contacted, 34 were reached, and 12 consented to participate. Reasons for non-participation included living in another country or being unavailable due to work commitments. The intended sample size was guided by recommendations for homogenous qualitative samples, which typically range from 7 to 19 participants to achieve thematic saturation [24, 25]. Data collection and preliminary analysis were conducted concurrently. Around the 10th interview, the research team noted that themes and concepts were beginning to repeat. The final two interviews were conducted to confirm that no new themes were emerging. Enrollment was stopped after 12 interviews, as the team agreed that thematic saturation had been achieved and no additional substantive information relevant to the study aims was being generated.

[24, 25]. Enrollment was stopped after *n* = 12 interviews because the research team, agreed that thematic saturation had been achieved. At this point, no new themes or substantive information relevant to the study’s aim were emerging from subsequent interviews.

### Data Collection

Interviews were conducted by a Spanish-bilingual researcher trained in qualitative methods (RHT) via telephone, lasting 15–30 min (mean = 19 min), and followed a semi-structured guide informed by the SEM framework [8, 9], which was introduced in the Background to contextualize the multiple levels influencing patient behavior. The SEM framework considers interacting levels that may affect engagement in tobacco treatment: individual (knowledge, attitudes, and skills), interpersonal (relationships with family, friends, and peers), organizational (clinical or workplace processes), community (social norms and neighborhood influences), and policy (laws, regulations, or institutional policies). The interview guide was designed to explore barriers and facilitators at each SEM level, with the aim of understanding the factors that influence engagement in tobacco treatment among Hispanic patients with cancer who currently use cigarettes (Table 1). Additionally, perceptions of the quality of discussions about tobacco use during routine clinical encounters with their oncology care providers were assessed. Interviews were audio recorded, transcribed verbatim, and de-identified for analysis. Data collection continued until thematic saturation was reached, meaning no new themes or subthemes arose from additional interviews [24].

### Data Analysis

Descriptive statistics were used to summarize patient characteristics, including demographic and cancer-related data, using medians and frequencies. Thematic analysis was employed to evaluate interview transcripts [26]. The analysis team consisted of the first author (RHT) and coauthors (LR, RCM, GBP, LC). For thematic analysis, a data matrix was created to facilitate deductive coding and thematic development, with each column representing an individual participant and each row corresponding to a predefined construct or domain from the SEM [9]. The first author reviewed all transcripts to identify statements relevant to the study aims, assigning each to the appropriate domain. Each statement was then coded with short descriptive labels that captured its meaning, ensuring consistency across transcripts; examples of these descriptive labels are provided as ‘codes’ in Table 2. This process was repeated for all transcripts. Subsequently, statements in the matrix and their associated codes were systematically categorized and reviewed across participants to identify primary themes and subthemes. This process was conducted collaboratively with the analysis team, who refined emerging themes through discussion. Participant quotations were translated from Spanish to English as part of this process. This rigorous approach resulted in an agreed-upon set of themes among all authors [27].

### Positionality and Reflexivity

The primary author, who led the data collection and analysis processes (RHT) is a Hispanic and Spanish-bilingual researcher with experience in psycho-oncology and tobacco use treatment. To account for potential influence of her professional background and personal experiences on data interpretation, reflexive practices were implemented throughout the project. To enhance cultural and analytical rigor, additional team members with experience working with Hispanic populations, cancer patients, and tobacco treatment (LR, RCM, GBP, LC) participated in the coding and thematic development process. During interviews, the researcher emphasized participant autonomy, ensured confidentiality, and encouraged participants to speak freely in their preferred language.

## Results

**Fig. 1:**
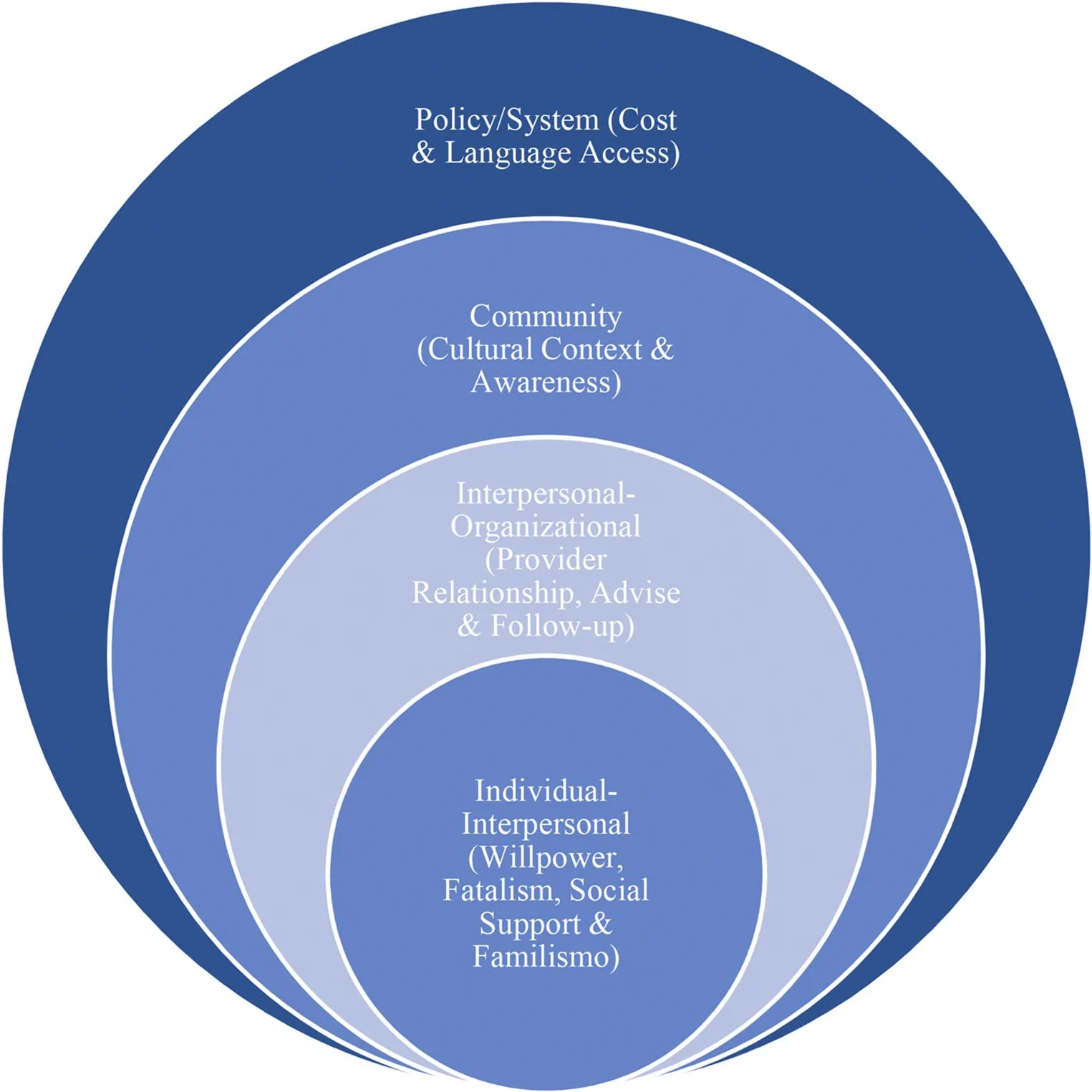
Adapted Socioecological Framework for Tobacco Treatment Engagement among Hispanic Patients with Cancer

Semi-structured interviews were conducted with 12 Hispanic patients referred to the embedded Tobacco Treatment Program (TTP). The median age was 65 years (IQR = 62–67.5). The sample consisted of 10 females and 2 males, with the majority (*n* = 10) preferring Spanish as their primary language. Participants were diagnosed with various cancers, including breast (*n* = 4), lung (*n* = 2), kidney (*n* = 1), metastatic adenocarcinoma (*n* = 1), pancreatic (*n* = 1), thyroid (*n* = 1), acute myeloid leukemia (*n* = 1), and cervix (*n* = 1). Half of the participants (*n* = 6) accepted tobacco treatment (Pts. 1, 3, 6, 7, 10, and 11), while the other half declined (Pts. 2, 4, 5, 8, 9, and 10). Those who accepted treatment had a lower median age (60 years) compared with those who declined (70 years). Among both groups, most preferred the use of Spanish for medical care. Table 2 presents the thematic development and coding structure of our qualitative analysis, organized according to the SEM to illustrate multilevel factors influencing Hispanic patients with cancer engagement with tobacco treatment programs. To better visualize the complex interplay of factors identified in our analysis, we developed an adapted SEM (Fig. 1). Unlike traditional linear models, this framework captures the overlapping nature of sociocultural values like *familismo* within individual factors, and the way provider persistence (Interpersonal-Organizational) can bridge the gap between community norms and systemic policy barriers.

### Themes

Thematic analysis guided by the SEM identified factors acting as barriers and facilitators to tobacco treatment engagement among Hispanic patients with cancer (Table 2). Four major themes emerged across the individual, interpersonal, organizational, community, and system/policy levels. Reaching saturation supports the adequacy of our sample size for capturing the key factors influencing tobacco treatment engagement in this population.

#### Theme 1: Socio-Cultural Drivers and the Psychological Barrier of Fatalism

- **Subtheme 1: Perceptions, Knowledge, Beliefs, and Emotional Influences.** Personal beliefs, knowledge, and emotional factors significantly influenced patients’ willingness to engage in tobacco treatment. Many held a strong belief that quitting was primarily a matter of willpower, viewing external aids like nicotine replacement therapy (NRT) as secondary; for them, motivation had to be internal: *“If the willingness is not within you*, *I don’t think a gum*, *or*, *a patch will… All three things need to come together: the gum*, *the patch*, *and your own willingness to quit smoking”* (Pt 1). Furthermore, psychological distress also played a major role, with many using smoking as a primary coping mechanism: ‘When I’m stressed from work or life, smoking is the only thing that calms me down even if I know it’s bad for me’ (Pt 2). For some, the vice was directly linked to trauma and isolation, becoming a necessary lifeline: *“I started smoking when I was experiencing domestic violence… the only thing that relaxed me was the cigarette*, *because I didn’t have family here”* (Pt 3). Beyond internal feelings, stigma powerfully shaped patient experiences. Some connected smoking to low socioeconomic status, viewing it as a normalized or “natural thing to have in the environment” “*Maybe I’m wrong*, *but in my opinion*, *we are people with low resources… and sometimes*, *well*, *they start smoking very early… it’s like a natural thing to have in the environment*” (Pt 8). Stigma combined with age-related fatalism—the belief that “the damage is done”—led to profound personal resignation, often intertwined with cultural identity: *“Well*, *how can I say? We Spanish speakers*, *we are stubborn about quitting something*, *really… what am I going to quit if that is the only thing I do? Listen. That’s how one thinks”* (Pt 1). This sense of futility was also reflected in imagined external judgment, as one patient imagined doctors thinking, *“at this stage of life*, *at 90 years old*, *it’s a bit tough”* (Pt 4). This judgment spurred some patients to actively counter stigma by using favorable medical reports to justify their smoking to family members: “*…and they were surprised because my lungs and bronchi were completely clean. Oh*, *okay. That also was a way of justifying myself to my family*” (Pt 9).
- **Subtheme 2: Social support and network.** Social environments (e.g., family and friends) emerged as both facilitators and barriers, either reinforcing persistent smoking behaviors or providing encouragement to quit and engage in tobacco treatment. Many participants described their social circles as a barrier to quitting, including friends and coworkers, who smoke and normalized smoking, making it difficult to quit. For example, “*The group I worked with were all smokers; during breaks*, *we would gather to smoke*” (Pt 8). Some noted a lack of support from friends and family who also smoke but have no intention of quitting. One patient shared, “*I have friends and family who smoke a lot and have no intention of quitting*” (Pt 9). Conversely, strong family encouragement was a key facilitator for others. One participant shared, “*My family always advised me to quit smoking*, *saying it wasn’t good… my children always told me to quit smoking*” (Pt 2). Another explained how their daughter’s influence helped them cut back: “*My daughter is the one who pushes me the most to quit smoking… when she comes to visit me*, *sometimes for a day*, *I smoke less because she doesn’t like the smoke*” (Pt 4). There was also a strong desire for peer support, particularly for groups that were culturally relevant (e.g., Spanish-speaking) and tailored to specific needs like those of patients with cancer. As one patient shared, “*If they had offered a Spanish-speaking group with cancer patients who smoke to manage stress*, *it would have benefited me*” (Pt 2).

#### Theme 2: Provider–patient relationship enhances engagement

- **Subtheme: Empathic communication, trust, and consistent follow-up with oncology and other healthcare providers.** Support from healthcare providers was seen as a critical factor in encouraging patients to engage with tobacco treatment. Patients often spoke about how oncology care provider persistence, consistent follow-up, and encouragement strengthened their quitting efforts. For example, one patient recalled, *“PROVIDER never stopped calling me*, *and I said yes. PROVIDER told me I had a lot of willpower*, *that I was going to make it”* (Pt 1). This persistence, combined with positive reinforcement and encouragement, empowered the patient to feel more capable of succeeding. Beyond just persistence, patients also highlighted the importance of empathic communication and trust in their relationships with oncology teams *“One of the doctors from MSK… I think it was the kidney doctor*, *Dr. PROVIDER*, *was the one that referred me… He said before I had my first surgery to have my left kidney removed*, *that I needed to stop smoking”* (Pt 5). When providers showed genuine support and respect, patients felt more motivated and confident about their ability to stick with treatment. One patient noted that their provider created a personalized and empathetic connection by linking the advice to a family history of cancer, stating, *“[The provider] told me that smoking wasn’t good for me*, *for my health*, *and even more so because I had two nieces who died of breast cancer”* (Pt 7). Patients also identified primary care providers as key players in promoting tobacco treatment engagement, emphasizing the need for consistent efforts in providing information and referrals. As one patient explained, “*Primary care doctors should do more to encourage their patients and refer them to places where they can get help”* (Pt 1).

#### Theme 3: Community and cultural contexts shaping tobacco use and support needs

- **Subtheme: Awareness of services, educational campaigns, and community events.** Community-based outreach strategies such as health talks, public events, and collaboration with community leaders were viewed as valuable avenues to facilitate treatment engagement. Participants emphasized the importance of outreach efforts to normalize quitting and provide culturally resonant support: *“Community leaders… present the program to them so they get involved and*, *in turn*, *engage the community”* (Pt 10). Several patients described receiving information about available services in culturally tailored ways that increased awareness and access. For example, one patient noted: *“My doctor gave me a brochure in Spanish with information about the hospital and its services”* (Pt 9), and another who had accepted treatment highlighted being informed about options: *“They explained to me that there are therapies and products like nicotine gum and patches”* (Pt 9). Patients also emphasized the value of broader public campaigns and community events to raise awareness. One suggested: *“It would be good to have more campaigns on television or at community events”* (Pt 8), while another recommended: *“Hold events where free products to quit smoking are distributed”* (Pt 8). Overall, participants underscored the importance of culturally and linguistically tailored education, particularly through talks and community sessions: *“Giving talks to people about the harm caused by cigarettes”* (Pt 2).

#### Theme 4: Structural, economic, and policy-level determinants constrain access; implementation and enforcement are key levers

- **Subtheme 1: Cost of tobacco treatment.** Patients frequently identified the cost of cessation resources as a barrier to engagement. One participant explained: *“If treatments were free or low-cost*, *more people would try them. Not everyone can afford treatments to quit smoking”* (Pt 8). Another reflected on the financial burden of nicotine replacement therapy: *“One time*, *I quit for almost two- or three-years using nicotine gum*, *but that gum cost 60 to 64 pesos”* (Pt 3). These observations suggest that high out of pocket treatment costs and limited insurance coverage, combined with limited structural support, may hinder cessation efforts and reduce participation in tobacco treatment programs.
- **Subtheme 2: Language barriers, need for interpreters, lack of culturally tailored care.** Participants underscored the importance of language access and culturally responsive services in supporting tobacco treatment engagement. For some, the availability of Spanish-language resources and interpreters made a critical difference: *“Also*, *the language (Spanish)*, *because I don’t speak English. These are the benefits of the program*, *you know; they are excellent”* (Pt 10). Others described the challenges of navigating care without adequate language support: *“With people who speak Spanish*, *because*, *for example*, *I try to express myself in Spanish*, *but it’s not that I fully understand it”* (Pt 4). Findings indicate that gaps in language access and culturally tailored care can unintentionally hinder participation in tobacco treatment programs, even when services are offered.
- **Subtheme 3: Weak enforcement of tobacco control laws and insufficient supervision.** Participants described tobacco control laws as often poorly enforced, which limited their effectiveness in supporting cessation. They noted that, although some areas prohibit smoking, people frequently circumvent these rules. As one participant explained, “*Even though there are places where smoking is not allowed*, *people always find ways”* (Pt 8), while another added, *“Even though there are places where smoking is prohibited*, *many people do not respect these rules”* (Pt 9). Another participant highlighted the mixed messages of partial restrictions: *“It’s okay*, *you don’t do it inside*, *but you can do it outside… They are restricting your consumption in one area*, *but at the same time*, *they are giving you the freedom to do it in another area”* (Pt 10). These observations suggest that inconsistent smoke-free enforcement and limited supervision at the community and policy levels may unintentionally facilitate continued tobacco use, even among individuals attempting to quit and participate in tobacco treatment.

### Multilevel Determinants of Treatment Engagement: A Comparative Analysis of Decisional Pathways.

The decision to accept or decline tobacco treatment was primarily determined by three thematic factors: age and fatalism, the patient’s interaction with the provider relationship, and the perceived role of willpower versus external support.

- **Individual-Level: Age-Related Fatalism and Perceived Benefit.** A clear distinction was observed demographically and psychologically. Patients who declined treatment had a higher median age (70 years) compared to those who accepted (60 years). This difference could be related to the presence of age-related fatalism, the belief that *“the damage is done”* (Pt 4). This psychological barrier may impact the perceived benefit of quitting post-cancer diagnosis, making treatment engagement seem futile. Conversely, acceptors, despite also coping with stress and nicotine dependency, demonstrated a lower degree of nihilism.
- **Individual-Level: Willpower Versus External Support.** Both groups adhered to the belief that quitting ultimately requires strong internal motivation. However, they differed in how they viewed professional aid. Decliners strictly adhered to the idea that motivation must be entirely internal, with one patient stating: *“If the willingness is not within you*, *I don’t think a gum*, *or a patch will… All three things need to come together: the gum*, *the patch*, *and your own willingness to quit smoking”* (Pt 1). This viewpoint often led them to reject treatment. Acceptors, while also believing in willpower, were willing to utilize external supports (pharmacotherapy, structured programs, and language access) as a necessary supplement to their personal resolve, effectively transitioning the struggle from a solitary fight to a supported process.
- **Interpersonal-Level: Provider Relationship and Trust.** The quality of the provider-patient relationship emerged as a critical tipping point. Acceptors appeared to be empowered by consistent, empathic follow-up and persistence from their oncology care team. For example, one patient who accepted treatment highlighted the provider’s tenacity: *“PROVIDER never stopped calling me*, *and I said yes”* (Pt 1). This sustained outreach helped overcome initial reluctance. Patients who declined, however, were characterized by an underlying distrust in professionals (as noted in the initial barriers list) and did not benefit from or were not receptive to persistent referral efforts.

## Discussion

The findings highlight key barriers and facilitators influencing Hispanic patients with cancer engagement with tobacco treatment. Participants reported a complex interplay of cultural beliefs, emotional factors, social environments, and structural barriers that affect their willingness and ability to participate in tobacco treatment. Key facilitators identified include the support of oncology healthcare providers and family members, while significant barriers include beliefs about tobacco and tobacco treatment, lack of culturally and linguistically tailored support services, and financial constraints. These results provide insight into the challenges Hispanic patients with cancer face when referred to a tobacco treatment and suggest potential areas for improvement in access and utilization of tobacco treatment programs in the context of cancer care.

Pre-existing beliefs regarding the value of tobacco treatment significantly influenced participants’ perceptions. Many treatment decliners emphasized the role of personal willpower in quitting, reflecting a cultural norm that may downplay the value of utilizing evidence-based treatment such as behavioral counseling and cessation medications. This perspective may also be related to low quitting self-efficacy [28]. Participants often attributed their persistent smoking to a lack of willpower or self-control and expressed doubt in their ability to quit and stay abstinent [11]. Furthermore, this could also point to a trend observed in Hispanic populations, where there is lower use of pharmacological support for smoking cessation [29].

Another crucial factor identified by treatment decliners was beliefs related to cancer and age, specifically the perception that participation in treatment would be futile, particularly among older patients. In our sample, age-related fatalism was present, where older patients who declined treatment (median age 70) often believed “the damage is done,” minimizing the perceived benefit of quitting at this stage. This psychological barrier is concerning, as national studies among older adults consistently show that quitting smoking at any age is beneficial for preventing second primary cancers and other adverse outcomes. While most older adults are advised to quit and express interest in doing so, only about one-third use proven cessation treatments, leading to a success rate of only 1 in 20 [30]. Therefore, tobacco treatment programs for Hispanic patients with cancer must actively counter fatalism by emphasizing the health benefits of quitting, even in later life, and by promoting the use of evidence-based treatments to enhance their chances of success.

Emotional distress, commonly experienced by patients with cancer, often led individuals to rely on smoking as a coping mechanism for stressors associated with illness and treatment, a pattern also identified in the general Hispanic community and other groups [11]. Additionally, the normalization of smoking within family and social environments emerged as a significant barrier to treatment engagement, as patients who declined treatment described social networks where smoking was not only prevalent but also socially accepted. Another finding consistent with the existing literature is that variability in insurance coverage and concerns about the out-of-pocket cost of treatment are common barriers to participating in tobacco treatment services. Other studies have identified that financial stressors may impact tobacco behavior among Hispanics [11, 14, 31]. Some patients may hesitate to disclose tobacco use or engage in cessation treatment due to concerns about insurance coverage or potential increases in premiums [32]. In the U.S., only a limited number of states currently prohibit tobacco-related insurance surcharges, which may heighten fears among patients about financial repercussions of reporting tobacco use. These concerns represent a structural barrier that could influence patients’ willingness to participate in treatment and should be considered when designing outreach and counseling strategies for Hispanic patients with cancer. Tailored patient education on available resources and social services is essential to effectively address negative social drivers of health (SDOH) that disproportionately affect Hispanic/Latino communities.

Another important engagement factor identified was the involvement of family members and support networks in encouraging participation of Hispanic patients with cancer in tobacco treatment. This can be understood through core Hispanic cultural values such as *familismo*. *Familismo*, or the strong emphasis on family connections, is central to many Hispanic/Latino communities, where family is the primary source of support, identity, and socialization [33]. For healthcare providers, it is essential to recognize the significant role that family plays in patients’ lives and to involve them in tobacco treatment engagement and planning. This highlights the need for culturally sensitive interventions that address both family support and behavioral change.

Healthcare provider involvement was recognized as a key facilitator of tobacco treatment engagement, with participants noting that consistent follow-up and positive encouragement from their oncologist played a significant role in their decision to seek tobacco treatment. However, barriers such as insufficient communication between providers and patients about available resources were also highlighted. This mirrors findings from studies that show Hispanic patients are less likely to receive advice about participating in tobacco treatment compared to other racial and ethnic groups [34]. That said, it is important to highlight that the use of an opt-out referral model has been shown to reduce disparities in access to tobacco treatment among Hispanic patients by facilitating equitable referrals and minimizing potential clinician bias [23].

Furthermore, patients expressed a strong preference for peer support groups, particularly those targeting the needs of Spanish-speaking patients with cancer. Providing peer support in a culturally appropriate environment could significantly enhance patient engagement and offer both emotional and practical support for individuals trying to quit smoking. Such support could foster a sense of community, reduce feelings of isolation, and create a space where patients feel understood and empowered. This need for culturally relevant peer support can be linked to cultural values, such as collectivism, which emphasizes the importance of community, family, and shared experiences. In cultures with collectivist values, individuals often place a higher priority on the well-being of the group rather than just the individual [35, 36]. As a result, incorporating peer support that aligns with these cultural values can create a more supportive, relatable, and effective environment for patients, ultimately potentially improving their chances of success in quitting smoking. Like *familismo*, which emphasizes family unity and support, collectivism can influence how individuals approach challenges and seek help within their social networks.

Given the time constraints and competing priorities faced by oncology providers, consistent counseling may not always be feasible. Tobacco Treatment Specialists (TTS) could help address this gap by providing dedicated behavioral counseling, follow-up support, and culturally tailored interventions [37]. Evidence from the Cancer Center Cessation Initiative (C3I) shows that programs staffed with TTSs identify strategies to enhance treatment access, patient connection, and tailored delivery in oncology settings, and that centers with greater tobacco treatment specialist resources demonstrate improved reach of evidence-based tobacco treatment [37, 38]. TTS could also facilitate peer-support groups, helping to strengthen the interpersonal level of support between patients and the healthcare system, ultimately enhancing engagement for patients who might otherwise face barriers due to provider limitations.

Community outreach efforts were identified as another key strategy for improving treatment engagement. Participants suggested that public awareness campaigns, local events, and the involvement of community leaders could help raise awareness about the dangers of smoking and the benefit of cessation even for older adults with a longstanding history of smoking. This aligns with studies showing that delivering tobacco treatment through community-based initiatives in Hispanic communities can be highly effective [39, 40]. These insights suggest that such interventions could be a powerful way to reduce stigma and nihilism and reach priority populations, such as Hispanic patients with cancer who smoke and their families. By creating a broader cessation support network, these efforts could help reduce the social isolation that many Hispanic patients with cancer face during their tobacco cessation journey.

Successful strategies to address these barriers have included culturally adapted interventions emphasizing family support, bilingual educational materials, community engagement, and trust in healthcare providers. Digital and mobile programs, such as text messaging interventions and apps, have shown promise in reaching Hispanics who smoke, while public health policies like graphic health warnings, price increases, and age restrictions on tobacco sales have been effective in promoting cessation and reducing initiation in this population [12].

### Limitations

Despite the encouraging findings, several limitations exist. The small sample size, limited to 12 participants, restricts the generalizability of the results. Additionally, the study was conducted at a single institution, which may not capture the full range of barriers faced by Hispanic patients with cancer across different healthcare settings. Future research should include a larger, more diverse sample of patients from various regions and institutions. Furthermore, studies exploring the effectiveness of community outreach programs and peer support groups, particularly for Spanish-speaking populations, could provide valuable insights into how these interventions can be optimized for engagement. Finally, addressing structural barriers such as insurance coverage and treatment costs will be crucial in ensuring equitable access to tobacco treatment for all patients.

### Recommendations

Based on the study findings, several recommendations are made to enhance tobacco treatment engagement among Hispanic patients with cancer. Our recommendations are highly consistent with those outlined by the American Lung Association in their *Hispanic/Latino Communities Toolkit*, which emphasizes the importance of improving access to smoking cessation programs and the need to integrate these resources into routine care, particularly for Hispanic communities, who often face barriers related to a lack of information and insufficient counseling from healthcare providers.

First, healthcare providers should routinely ask about smoking status and inform all patients who report current smoking about available tobacco cessation services in their preferred language. This should include not only information about local programs but also state- or federally funded, free resources. Ensuring that patients are aware of all the resources available to them can help overcome practical barriers related to lack of awareness and accessibility, particularly among those who may be unfamiliar with available programs due to language barriers.

Second, the establishment of Spanish-speaking peer support groups is strongly recommended. These groups would provide a culturally relevant space for patients to share experiences and receive emotional support in their native language. Peer support has been shown to be a powerful motivator for smoking cessation, and providing these groups within a culturally familiar setting could enhance engagement, particularly for those who may feel isolated in their quitting journey. Beyond informal peer support, professionally led group treatment in Spanish could also be offered. Such groups can provide the dual benefit of peer support and structured, evidence-based counseling from a trained bilingual facilitator, which may be especially important given patients’ expressed desire for more provider-supported treatment.

Third, it is essential to offer accessible and culturally relevant educational sessions on the specific benefits of quitting tobacco following a cancer diagnosis. These sessions should be tailored to the specific needs of Spanish-speaking patients, incorporating cultural norms and practices. By providing such sessions, healthcare providers can better address patients’ concerns and motivations, making it more likely for them to engage with tobacco treatment programs.

Fourth, improving the dissemination of educational materials is critical. Written materials, as well as digital content such as mobile applications, should be provided in Spanish to ensure accessibility. This could include information on the benefits of quitting, practical strategies to manage cravings, and available support programs. Clear, accessible, and culturally tailored resources can help bridge the information gap and encourage patient participation in treatment.

Fifth, training Spanish-speaking providers in tobacco use assessment and treatment is crucial. By enhancing the skills of Spanish-speaking providers in assessing tobacco use and providing appropriate treatment options, communication barriers can be reduced, and trust can be built with patients. This would also enable better support for patients throughout their cessation journey.

Sixth, expanding access to telehealth cessation support should be considered, particularly for patients who have difficulty attending in-person sessions due to logistical challenges or health concerns. Offering virtual meetings through telehealth platforms can make participation in cessation programs more convenient, allowing patients to attend sessions from the comfort of their homes while still receiving the support and guidance they need to quit smoking. At a minimum, programs should ensure the availability of Spanish interpretation services for phone and video telehealth visits, as not all organizations may have Spanish-speaking clinicians. Providing language-concordant or interpreted care is essential to ensure equitable access and effective communication in tobacco cessation services.

Finally, recognize the role of family dynamics in tobacco use and recovery by incorporating family-centered approaches into cessation programs. This may include involving family members in counseling sessions, providing education on how to support a loved one’s quit attempt, and addressing relational factors that may impact smoking behaviors. Table 3 lists key resources available in Spanish to support tobacco cessation, including culturally tailored programs, educational materials, and multilingual counseling options for Hispanic patients.

## Figures and Tables

**Table 1:** Sample Interview Questions Aligned with the Social Ecological Model

Level	Examples of Interview Questions

Individual	When you hear the words “tobacco treatment”, what comes to mind? How do you feel about it?
Interpersonal	Do you feel comfortable discussing tobacco cessation options with healthcare providers? If not, what barriers do you face in communicating about your smoking or treatment preferences?
Organizational	What efforts have you observed from MSK to assist Hispanic patients with cancer who smoke in accessing tobacco treatment?
Community	How does tobacco use manifest in your community?
Policy and Systems	How might the cost of nicotine replacement and tobacco treatment influence your interest in participating in MSK’s program?

**Table 2:** Thematic Development and Coding Structure Guided by the Socioecological Model

Theme	Subtheme	Codes	SEM Level

**Socio-Cultural Drivers and the Psychological Barrier of Fatalism**	Perceptions, Knowledge, Beliefs, and Emotional Influences	Willpower, Self–Efficacy, and Beliefs; Emotional Regulation & Mental Health; Health Perceptions	Individual/Interpersonal
	Social Support and Networks	Social Influence; Family & Friend Support; Peer Connection	
**Provider–Patient Relationship Enhances Engagement**	Empathic Communication, Trust, and Consistent Follow-Up	Provider Relationship/Interaction; Communication; Advice; Follow Up	Interpersonal/Organizational
**Community and Cultural Contexts Shaping Tobacco Use and Support Needs**	Awareness of Services, Educational Campaigns, Community Events	Education; Promotion; Services Knowledge	Community
**Structural, Economic, and Policy-Level Determinants Constrain Access; Implementation and Enforcement are Key Levers**	Cost of Treatment Language Barriers and Lack of Culturally Tailored Care Weak Enforcement of Tobacco Control Laws	Cost; Access; Language; Policy Enforcement	Organizational/System & Policy

**Table 3:** Resources for Tobacco Cessation Support in Spanish

Resource Name	Description	Type	Link

MSKCC Tobacco Treatment Program	Provides culturally adapted materials, counseling options, and referral support in Spanish for patients with cancer.	Virtual, Print	*Guía para el tratamiento del tabaquismo: para pacientes y sus familias | Memorial Sloan Kettering Cancer Center*
Addressing Tobacco Use in Hispanic or Latino Communities Toolkit (American Lung Association)	Toolkit emphasizing access to smoking cessation programs, culturally relevant patient education, and integration into routine care.	Print, Online Toolkit	ALA Hispanic/Latino Toolkit
CDC Quit Smoking in Spanish	Offers Spanish language fact sheets, and self-help resources.	Online, Print	CDC Deje de Fumar
Smokefree Español (NCI)	Bilingual website and text message program providing tailored support for Spanish speakers to quit smoking.	App, Text Messaging, Web	Smokefree Español
National Quitline (1-855-D-JELO-YA)	Free and confidential quitline offering counseling in Spanish.	Phone	1-855-335-3569

## Data Availability

Data supporting the findings of this study are available from the corresponding author upon reasonable request.
